# The role of acculturation in the process of advance care planning among Chinese immigrants: A narrative systematic review

**DOI:** 10.1177/02692163231179255

**Published:** 2023-06-13

**Authors:** Tingting Zhu, Diah Martina, Agnes van der Heide, Ida J Korfage, Judith AC Rietjens

**Affiliations:** 1 Department of Public Health, Erasmus MC, University Medical Centre Rotterdam, Rotterdam, The Netherlands; 2 Department of Medical Oncology, Erasmus MC Cancer Institute, University Medical Centre Rotterdam, Rotterdam, The Netherlands; 3 Division of Psychosomatic and Palliative Medicine, Department of Internal Medicine, Universitas Indonesia, Jakarta, Indonesia; 4 Dr. Cipto Mangunkusumo National General Hospital, Jakarta, Indonesia; 5 Department of Design, Organization and Strategy, Faculty of Industrial Design Engineering, Delft University of Technology, Delft, The Netherlands

**Keywords:** Asian continental ancestry group, advance care planning, acculturation, emigration and immigration, systematic review

## Abstract

**Background:**

Acculturation is the process of two different cultures coming into contact. It is unclear how acculturation influences Chinese immigrants’ engagement in advance care planning due to the complexity and multifaceted nature of both acculturation and advance care planning.

**Aims:**

To synthesize evidence regarding the role of Chinese immigrants’ acculturation in their engagement in advance care planning.

**Design:**

Systematic mixed-method review, registered in PROSPERO (CRD42021231822).

**Data sources:**

EMBASE, MEDLINE, Web of Science, and Google Scholar were searched for publications until January 21, 2021.

**Results:**

Twenty-one out of 1112 identified articles were included in the analysis. Of those 21 articles, 17 had a qualitative design and 13 originated from the United States. Three of four quantitative studies reported that higher acculturation levels were associated with better knowledge or higher rate of engagement in advance care planning. Analysis of qualitative studies showed that Chinese immigrants’ engagement in advance care planning was associated with their: (1) self-perceived cultural identity (native or non-native); (2) interpretation of filial piety (traditional or modern); and (3) interpretation of autonomy (individual or familial). To facilitate their engagement, Chinese immigrants prefer an implicit approach, non-family-related initiators, contextualization advance care planning in Chinese culture and using Chinese language.

**Conclusion:**

Chinese immigrants’ willingness to engage in advance care planning varied with their acculturation level. To engage them in advance care planning, we recommend adapting the introduction of advance care planning to address people’s perceptions of their cultural identity, filial piety, and autonomy, as well as their preference for certain approach, initiator, context, and language.

**What is already known about the topics?**

- Chinese immigrants are the largest and most rapidly growing minority group in Western countries. When compared with non-immigrant Western populations, they are less likely to engage in advance care planning and more likely to receive life-sustaining treatments.
- Both Chinese traditional culture and the mainstream culture of host countries impact Chinese immigrant’s perception of death and preferences regarding serious illness conversations.
- Literature suggests that the level of acculturation is an essential factor to consider in Chinese immigrants’ advance care planning.

**What this paper adds?**

- This study demonstrates that acculturation affects Chinese immigrants’ self-perceived cultural identity as well as their interpretation of filial piety and/or autonomy, which are key cultural aspects influencing their perceptions of and willingness to engage in advance care planning.
- This study suggests that engaging Chinese immigrants in advance care planning can be facilitated by: (a) an implicit approach; (b) initiation by non-family members; (c) contextualization in a Chinese cultural context; and (d) the use of Chinese language.

**Implication for practice, theory, or policy?**

- Being aware of the multiple elements of acculturation and its continuum can help healthcare professionals understand the diversity in Chinese immigrants’ engagement in advance care planning.
- In view of varying acculturation levels among Chinese immigrants, healthcare professionals should avoid stereotyping their attitudes and preferences regarding advance care planning in clinical practice.
- Facilitating Chinese immigrants’ advance care planning requires applying culturally sensitive approaches (e.g. implicit communication strategies, non-family-related initiators, contextualization of foreign concepts, and using the Chinese language).

## Introduction

Advance care planning enables individuals to define goals and preferences for future medical treatment and care, to discuss these goals and preferences with family and healthcare professionals, and to record and review these preferences if appropriate.^1^ Advance care planning has been proven to increase patient and surrogate satisfaction with communication and care. It can also reduce the stress and anxiety that loved ones may feel if they are unsure of patients’ wishes.^2^ Lack of such planning for end-of-life care might lead to a higher rate of intensive care (e.g. life-sustaining treatment implementation, intensive care admission) and worse quality of death.^3,4^ At its core, advance care planning is based on a number of principles and values rooted in mainstream Western bioethics, such as patient autonomy, decision-making based on informed consent, truth-telling and control over the dying process.^5^

Advance care planning is a relatively new concept in mainland China, where most people are still reluctant to talk about death, as they consider it a taboo topic.^6^ These beliefs and social norms including the core value of collectivism in traditional Chinese society,^7,8^ differ from those of Western societies. Previous studies show that these cultural factors may influence engagement in advance care planning among Chinese people.^9–11^ Patients’ wishes regarding end-of-life care have rarely been discussed, and the general public has limited understanding of and willingness to engage in advance care planning.^12–14^

Migrating to a society that is more open to advance care planning may influence one’s perspectives and practice regarding how to tailor their preferred medical care and the decision-making process.^15^ Chinese immigrants constitute the largest immigrant population worldwide, with a large portion settling in North America, Western Europe, and Australia.^16^ The majority of Chinese immigrants came from Mainland China, followed by Taiwan, Hong Kong, and Macao.^17^ Chinese immigrants came to prominence as a rapidly growing group who are less likely to engage in the advance care planning process and more likely to receive aggressive life-sustaining treatments at the end-of-life when compared with non-immigrant Western populations.^3,4,18^

A recent review found that Chinese immigrants’ advance care planning engagement could potentially be influenced by their acculturation.^19^ Acculturation is the process of two different cultures coming into contact.^20^ As a result of this process, Chinese immigrants’ attitudes and behaviors toward advance care planning may reflect the values of both Western and Eastern cultures. Studies in Western countries^1^ and Asia^21^ have provided insight regarding how to better facilitate one’s engagement in advance care planning within the Western and Asian culture, but little is known about advance care planning in Chinese immigrants who may be holding on to their heritage culture and adapting to the culture of their host country. However, studies on advance care planning among Chinese immigrants were conducted in different host countries in different settings, using various conceptualizations of acculturation (e.g. birth place, residence time, generation, language proficiency, etc.),^22–25^ and different definitions of advance care planning.

To better understand how advance care planning can be better delivered to this group, it is essential to understand the role of acculturation in its process. We aimed to systematically synthesize evidence pertaining to the Chinese immigrants’ acculturation and its role in their engagement in advance care planning.

## Methods

This mixed-method narrative systematic review was reported according to the Preferred Reporting Items for Systematic Reviews and Meta-Analyses (PRISMA) 2020.^26^ The study protocol had been registered in the International Prospective Register of Systematic Reviews (PROSPERO: CRD42021231822). A convergent segregated approach was used for the synthesis and integration of this systematic review.^27^ Our research question aims to address two different aspects associated with the role of acculturation in Chinese immigrants’ advance care planning: 1. the association between acculturation and Chinese immigrants’ advance care planning. This association has been addressed through quantitative studies, and 2. how Chinese immigrants experience or perceive advance care planning in the context of acculturation. The experience and perception have been answered through qualitative studies.

## Data sources and searches

With the aid of a biomedical information specialist, we conducted a systematic literature search to include all English-language publications on advance care planning in the following electronic databases: Embase.com (1971-), MEDLINE ALL Ovid (1946-), Web of Science Core Collection (1975-), and Google Scholar from inception to January 21, 2021 (date last searched).

We used tailored search terms for each database, using thesaurus terms (Emtree and MeSH) where applicable. Supplemental Table 1 shows the search terms for each database. The search terms did not only contain words for advance care planning and advance directives but also included terms pertaining to end-of-life decision-making.^21,28^ Additionally, to ensure inclusivity, one author (T.Z), who has a Chinese background, conducted searches in two Chinese electronic databases, CNKI (1999-) and WanFang (1998-), using search terms including immigrants, advance care planning, advance directive, and living will. Moreover, to ensure a comprehensive search, we manually searched the reference lists from relevant studies to confirm whether important studies that met our inclusion had been missed.

## Study selection

Studies were included based on the following inclusion criteria:

(1) The study should be an original empirical study published in English in a peer-reviewed journal;
(2) The study should be about Chinese immigrants,^29^ defined as adults whose self-identified ethnicity was Chinese, older than 18 years, born inside or outside the host country. We included studies where participants were Chinese immigrants (including older adults, their family members, and Chinese community leaders, etc.), or those involved in their care for medical decision-making (including health care or social care professionals, or medical interpreters, etc.). We restricted the host country of Chinese immigrants to the top 25 countries of the quality of death index 2015^30^ (excluding Asian countries and districts), to better understand the impact of social and cultural norms on advance care planning for Chinese immigrants in countries with more developed palliative care systems.
(3) The study should address advance care planning. We defined advance care planning as: (i) activities the authors had labeled as “advance care planning,” (ii) activities involving patients, family and/or healthcare professionals discussing patients’ preferences/wishes for future medical treatment and/or care, or (iii) activities that involve documentation processes of patients’ preferences, including the appointment of a personal representative and writing an advance directive.^1^
(4) We defined acculturation as those phenomena which result when groups of individuals having different cultures come into continuous first-hand contact, with subsequent changes in the original culture patterns of either or both groups.^15^ In the context of healthcare, acculturation involves tailoring societal norms and healthcare ideals of the host society to meet individuals’ cultural preferences.

In our study, we operationalized acculturation by adopting the elements of acculturation that have been recognized as dimensions in established multidimensional measurement tools. We developed conceptualizations for each element based on the descriptions provided by the corresponding measurement tools and the content of their items. We used the concept of each element to extract data that aligned with each element or with the broader definition of acculturation. After data extraction and its alignment with the appropriate element, TZ and DM jointly ensured that all the extracted data aligned with the intended meaning of the element or the broader definition of acculturation. Any disagreements between the reviewers were resolved through discussion.

The elements of acculturation included but were not limited to: language use/preference, social affiliation, daily living habits, communication style, cultural identity/pride, perceived prejudice/discrimination, generational status, and cultural tradition/value orientations.^31^

Two authors (TZ, DM) screened titles and abstracts for eligibility and then reviewed full-text articles independently. If necessary, disagreements were discussed and resolved with all authors. References were managed using Endnote bibliographic software version X9©.^32^

## Quality assessment and data extraction

Two reviewers (TZ, DM) independently assessed the methodological quality of included studies using the QualSyst tool.^33^ Qualitative studies and quantitative studies were evaluated using a validated 10-item evaluation checklist and a validated 14-item evaluation checklist respectively. Summary scores for individual studies were categorized as strong (score of >0.80), good (0.70–0.80), adequate (0.51–0.69), or limited (⩽0.50). Disagreements were resolved via constant comparison and discussion.

A tailored data-extraction form was developed by TZ. After piloting by JR, dual data extraction was conducted by TZ and DM independently, which included: (a) study characteristics; (b) the perspectives of Chinese immigrants and those involved in their care on advance care planning; (c) their understanding and acceptance of the concept and necessity of advance care planning; (d) their opinions on the role of proxy decision-makers and communication processes; (e) Chinese immigrants’ willingness to engage in advance care planning; (f) the underlying motivations of Chinese immigrants’ engagement in advance care planning. We extracted data from (b-e) that reflect our conceptualizations for elements or the broader definition of acculturation. The extracted data was then reviewed by both TZ and DM to reach a consensus.

## Data synthesis and analysis

We conducted a parallel-results convergent synthesis^34^ analyzing both quantitative and qualitative evidence was conducted to explore whether and how Chinese immigrants’ acculturation influences their engagement in advance care planning. We narratively synthesized quantitative data according to the Guidance on the Conduct of Narrative Synthesis in Systematic Reviews^35^ by textual description, tabulation, and translation to integrate relationships based on the research question. The qualitative data were analyzed on the basis of Boeije’s procedure^36^ for thematic content analysis.^37^ First, a dual open coding to generate initial codes from the original text was conducted by TZ and DM independently. We then collated all codes into themes through constant discussions. All potential themes were reviewed and integrated with coded data and the data set constantly before defining and naming each theme. This process was facilitated through continued discussions with all authors. Qualitative analysis software (NVivo 12 Pro) was used to organize all qualitative data. Disagreements in data analysis process were settled by consensus.

## Results

### Study characteristics

**Figure 1.**
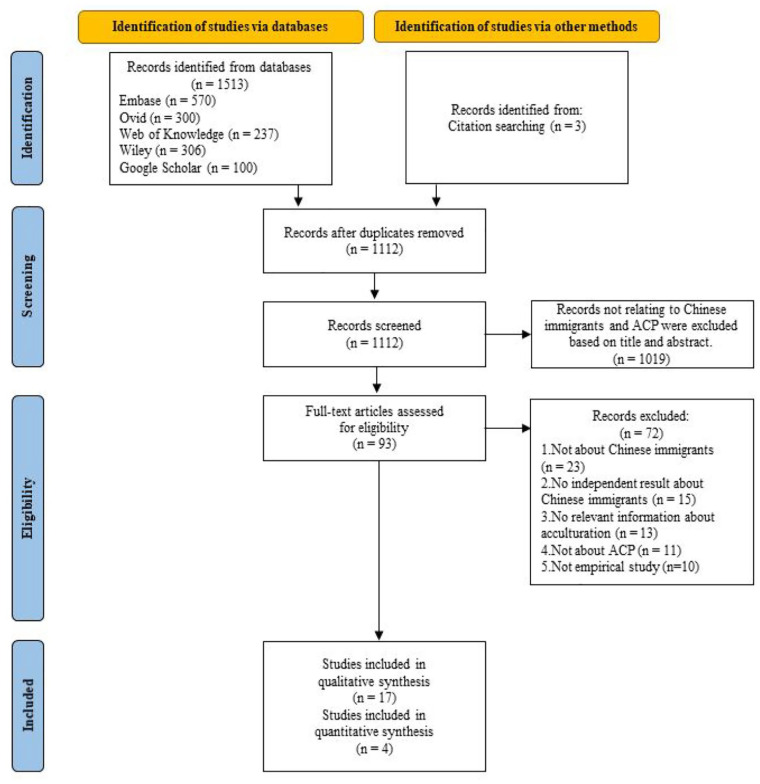
PRISMA flowchart for study selection.

Through our systematic search (*n* = 1513) and a manual search of reference lists (*n* = 3), we identified 1516 potential studies (Figure 1). After de-duplication, 1112 studies remained, which were then screened on the basis of their titles and abstracts. We excluded 1019 studies in which no Chinese immigrants participated or that had not studied advance care planning. We then screened the full texts and excluded 72 articles. Reasons for exclusion can be found in the flowchart (Figure 1). Twenty-one studies were ultimately included,17 of which used qualitative methods (Table 1). A majority of the studies (*n* = 13) had been conducted in the United States.^22–25,38–46^ Most studies (*n* = 16) enrolled participants from outside the clinic/hospital.^22,24,25,38–41,43–45,47–52^ Three studies enrolled healthcare professionals together with Chinese immigrants (as the targeted participants).^38,39,53^ The term advance care planning was used in four studies.^25,41,43,51^ Sixteen studies conceptualized advance care planning as a process of conversations about personal preferences.^24,25,38,39,41–44,46–51,53,54^ Cultural identity (*n* = 11) and cultural value (*n* = 10) were the most-frequently studied elements of acculturation. Methodological quality was categorized as being strong in seven studies, good in eight, adequate in three, and low in three (Supplemental Tables 2 and 3).

**Table 1.** Overview of included studies (*n* = 21).

Study characteristics	Qualitative study *N* = 17 (%)	Quantitative study *N* = 4 (%)
Host country		
The United States of America	9 (53)	4 (100)
Canada	4 (23)	-
Australia	2 (12)	-
The United Kingdom	1 (6)	-
New Zealand	1 (6)	-
Setting		
Community	13 (76)	3 (75)
Inpatient care	3 (18)	-
Outpatient care	1 (6)	1 (25)
Number of Chinese immigrants in the study		
0–100	17 (100)	-
100–500	-	4 (100)
Type of participants in the study		
Chinese immigrants^a^	15 (88)	4 (100)
Other^b^	2 (12)	-
Term related to advance care planning		
End-of-life care discussion	4 (24)	-
End-of-life decision making	4 (24)	-
End-of-life discussion	3 (17)	-
Advance care planning	3 (17)	1 (25)
Advance directive	2 (12)	3 (75)
End-of-life planning	1 (6)	-
Conceptualization of advance care planning		
Advance care planning as a completion of documents	2 (12)	3 (75)
Advance care planning as a process of discussion of personal preferences	15 (88)	1 (25)
Elements of acculturation as described in the study^c^		
Cultural value	10	-
Cultural identity/pride	8	3
Communication style	8	-
Cultural tradition	7	-
Language use/preference	6	3
Generational status	3	1
Social affiliation/family socialization	2	3
Perceived prejudice/discrimination	2	-
Level of acculturation	-	-
Daily living habit	-	3
Years of residence	-	1
Birthplace	-	1

a Chinese immigrants including patients, family members, community residents, community leaders.

b Others: Healthcare professionals with the experience in providing care to Chinese immigrants were included in three studies^38,39,53^;Chinese speaking medical interpreters.^42^

c Studies studied more than one element of acculturation.^31^

### Narrative synthesis of quantitative findings

We identified four quantitative studies, which all originated from the United States (Table 2). Two studies reported that higher acculturation levels were associated with better knowledge or higher odds of recording advance care planning.^22,24^ One study showed that being born in the United States was associated with a higher likelihood of advance care planning contemplation and discussion compared to being born in China.^25^ One study^23^ on underserved Chinese immigrants, whose levels of acculturation were low, showed that the acculturation level was not associated with awareness and documentation of advance care planning.

**Table 2.** Characteristics and key findings of quantitative studies.

No.	Author, year (Host country)	Research aims	Setting	Health status (of Chinese immigrants)	Number of participants (Type of participants)	Element of acculturation measured	Operationalization of advance care planning	Findings
1	X Gao, 2015 (the United States)^22^	To describe knowledge of AD and preferences regarding EOL care communication, decision making, and designation of surrogates and the role of acculturation variables in AD awareness.	Community	Non-specific	385 Chinese immigrants	- Level of acculturation 1. Language preferences 2. Daily living habit 3. Social affiliation 4. Cultural identity - Years of residency	Advance directives	The acculturation levels and years of residency in the US were statistically significant associated with the awareness of advance care directives
2	L Dhingra, 2020 (the United States)^23^	To describe attitudes and beliefs concerning ACP in older, non-English-speaking Chinese Americans in a medically-underserved urban region	Primary healthcare center	Patients with chronic conditions	179 Chinese immigrants	- Language preferences - Daily living habit - Cultural identity/pride - Social affiliation - Generational status	Advance directives	The acculturation level of participants was low. No significant associations between the level of acculturation and awareness and documentation of advance care directives
3	Y Pei, 2021 (the United States)^25^	To examine how immigrant status and family relationships are associated with ACP engagement and EOL preference in burial planning among older Chinese Americans.	Community	Non-specific	430 Chinese immigrants	Generational status	1. End-of-life care discussion with family 2. Burial Planning	The US-born Chinese Americans were associated with a higher likelihood of ACP contemplation and ACP discussion than the foreign-born.
4	K Wang, 2021 (the United States)^24^	To examine the factors of AD completion among older Chinese Americans	Community	Non-specific	435 Chinese immigrants	- Level of acculturation 1. Language preferences 2. Daily living habit 3. Social affiliation 4. Cultural identity	Advance directives	The level of acculturation was significantly associated with completion of advance care directives (the odds of completion at least doubled with one additional unit of acculturation)

### Thematic content analysis of qualitative findings

**Figure 2.**
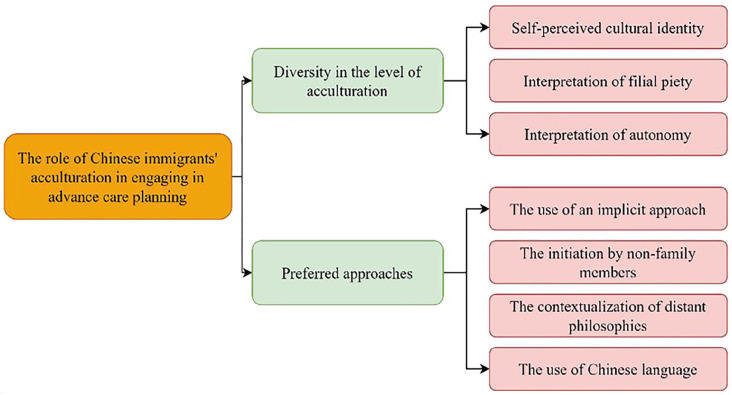
Coding tree of the role of Chinese immigrant’s acculturation in their engagement in advance care planning.

We identified two overarching themes regarding the role of Chinese immigrants’ acculturation in their engagement in advance care planning: (1) Diversity in the level of acculturation and (2) Approaches to advance care planning as recommended by Chinese immigrants or those involved in their care or medical decision-making (Figure 2, Table 3). Chinese immigrants’ acculturation influences their cultural identity, interpretation of filial piety and autonomy. To better facilitate their engagement in advance care planning, four recommended approaches were identified: (1) the use of an implicit approach, using culturally sensitive communication strategies, integrating advance care planning in clinical routines and community activities; (2) the initiation of advance care planning by non-family members; (3) the contextualization of advance care planning to make sense of distant philosophies; and (4) the use of Chinese language. We will describe these findings in more detail below.

**Table 3.** Characteristics of qualitative studies.

	Author, year (Host country)	Research aims	Study design	Setting	Health Status (of Chinese immigrants)	Number of participants (Type of participants)	Conceptualization of advance care planning	Elements of acculturation
1	KL Braun,1996 (the United States)^45^	To explore death beliefs in Asia groups	Semi-structured interviews	Community	Non-specific	5 Chinese immigrants	Advance directives	Generational status
2	KL Braun,1997 (the United States)^40^	To explore approaches to death and dying in Asian Americans	Semi-structured interviews	Community	Non-specific	5 Chinese immigrants	Advance directives	Generational status
3	KW Bowman, 2001 (Canada)^47^	To describe Chinese immigrants’ views about end-of-life decisions	Semi-structured interviews	Community	Non-specific	40 Chinese immigrants	End-of-life decision-making	1. Cultural value (Not being burdensome) 2.Cultural tradition (Talking death a taboo; Harmony) 3.Generation status 4.Cultural value (Filial piety)
4	AG Yick, 2002 (the United States)^44^	To describe Chinese Americans’ beliefs and practices regarding death and dying	Focus groups interviews	Community	Non-specific	13 Chinese immigrants (7 social worker, 6 pastors and religious leaders)	End of life discussion	Communication style
5	J Chan, 2005 (the United States)^46^	To investigate the clinical environmental social and cultural factors influencing the care of the terminally ill nursing home residence	Event analysis; Participation observation; In-depth interviews	Nursing home	Terminally ill Chinese older patients	34 Chinese immigrants	End-of-life decision-making	1. cultural identity/pride 2. language use/preference 3. cultural value (Family-center decision making)
6	G Bellamy, 2013 (New Zealand)^54^	To explore the views of healthcare staff working in New Zealand	Joint interviews; Focus groups interviews	Hospital	Not applicable	2 healthcare professionals (out of 80, others were not interacting with Chinese immigrants)	End-of-life decision-making	Cultural value (Family-centered decision making)
7	ML Fang, 2015 (the United Kingdom)^48^	To explore the end-of-life beliefs, values, practices, and expectations of a select group of harder-to-reach Chinese women living in England	Semi-structured interviews	Community	Non-specific	11 Chinese immigrants	End-of-life discussion	1. Social affiliation 2. Cultural value (filial piety) 3. Perceived discrimination 4. Language use/preference
8	LS Nielsen, 2015 (Canada)^53^	To explore discursive tensions present in home care policies when providing palliative home care to Chinese immigrants.	Focused ethnographic study	Home care	Chinese immigrants with advance cancer	8 Chinese immigrants (4 Chinese immigrants, 4 primary family caregivers); 15 healthcare professionals	End-of-life care discussion and planning	1.Cultural identity 2.Language use/preference
9	J Yonashiro-Cho, 2016 (the United States)^43^	To explore the knowledge, attitudes, and preferences of older Chinese Americans toward ACP	Focus groups interviews	Community	Non-specific	34 Chinese immigrants	Process of discussion on preferences	1.Cultural tradition (Parents works things together) 2.Cultural tradition (Talking about death as taboo) 3.Communication style
10	MC Lee, 2017 (the United States)^41^	To explore behavioral, normative, and control beliefs in the discussion of advance care planning (ACP) among older and younger Chinese Americans.	Observation of group interaction; Semi-structured interviews	Community	Non-specific	60 Chinese immigrants (30 older generations, 30 younger generations)	Process of discussion on preferences	1.Cultural identity/pride 2.Language use/ preference 3.Cultural value (Not being burdensome to the family) 4.Generation status 5.Cultural value (Filial piety) 6.Cultural tradition (Social harmony) 7.Communication style
11	SS Yap, 2017 (Australia)^51^	To identify factors that influence the engagement of Chinese Australians with advance care planning	Semi-structured interviews	Community	Non-specific	30 Chinese immigrants	Process of discussion on preferences	1.Perceived prejudice 2.Cultural value (filial piety) 3.Language use/preference 4.Cultural identity
12	HL Chi, 2018 (the United States)^38^	To explore older Chinese Americans and adult children’s communication preferences and optimal timing for HCPs to initiate the EOL care discussions	Semi-structured interviews	Community	Non-specific	23 Chinese immigrants (14 older Chinese adults, 9 adult children); 7 healthcare professionals	End-of-life care discussion	1. Cultural identity/pride 2. Cultural tradition (Trust authority; Parents work out things together) 3. Communication style
13	HL Chi, 2018 (the United States)^39^	To explore HCP communication strategies to initiate EOL care discussions with older Chinese Americans and their families	Field observation; In-depth interviews	Community	Non-specific	23 Chinese immigrants (14 older Chinese adults, 9 adult children); 7 healthcare professionals	End-of-life care discussion	1. Cultural tradition (Talking about death as taboo) 2. Communication style
14	C Lou, 2020 (Canada)^49^	To explore the generalizability of the empiric Dignity Model to Chinese Canadians an immigrant population influenced by both Western and Asian values. The study will explore how dignity is culturally mediated	focus groups interviews	Community	Non-specific	31 Chinese immigrants	End-of-life medical decision-making	1.Cultural value (Filial piety) 2.Cultural identity
15	MD Silva, 2020 (the United States)^42^	To develop insights from medical interpreters about their role when interpreting discussions regarding EOL issues, identify practices interpreters perceive as helping to improve or hinder patient-provider communication, and obtain suggestions on how to improve communication during EOL conversations with Spanish-speaking and Chinese-speaking patients	Semi-structured interviews	Hospital	Not applicable	5 medical interpreters	End-of-life conversation	1.Communication style 2.Cultural tradition (Chinese philosophy)
16	WY Wang, 2020 (Australia)^52^	To explore the role of stakeholders in constructing new socio-cultural narratives of advance care planning in the Chinese community in Australia	Semi-interviews; Field observation	Community	Non-specific	40 Chinese immigrants (16 Chinese community stakeholders, 24 Chinese individuals)	End-of-life planning	1.Communication style 2. Social affiliation 3. Language use/preference
17	W Zhang, 2020 (Canada)^50^	To understand who are more willing to talk about death? Or is the cultural taboo of death disintegrating?	Focus groups interviews	Community	Non-specific	46 Chinese immigrants	End-of-life discussion	1.Cultural identity 2.Cultural tradition (Talking about death as taboo) 3.Communication style 4.Cultural identity

### Diversity in the level of acculturation

Chinese immigrants’ willingness to engage in advance care planning was influenced by their self-perceived identities, interpretation of filial piety, and interpretation of autonomy.

1a. Differences in self-perceived cultural identities

Chinese immigrants varied in the way they perceived their own cultural identities. Our finding shows that immigrants’ self-perceived identities range across a continuum.

At one end of the continuum, Chinese immigrants considered themselves as Chinese descents and therefore they held on tightly to the traditional Chinese culture. They believed that advance care planning is incompatible with their cultural belief since death-related conversations are taboo.^40,41,44–47^

*“I don’t think Chinese people will like to do this [advance directives]. People will avoid topics that make them feel negative.”*

*(Older Chinese adult)* ^47^

At the other end of the continuum, Chinese immigrants considered themselves part of the host society and adopted the host country’s cultural norms. They reported that engaging in advance care planning in the host country aligns with their newly adopted cultural values.^49–51^

*“We are very open-minded that we sit around and talk about death. We would not say, don’t talk with me about those unlucky matters. But when you are in Hong Kong or mainland China, do not mention it.”*

*(Older Chinese immigrant)* ^50^

1b. Differences in the interpretation of filial piety

According to the traditional Chinese interpretation of filial piety, a filial child is expected to “fight with all strength” in pursuing measures to prolong their parents’ life. According to some Chinese immigrants, children agreeing to and supporting parents’ wishes to withhold or withdraw life-sustaining treatments may be considered not being filial.^47–49,53^

*“The children don’t dare to decide not to save their parent’s life, they will feel uneasy.”*

*(older Chinese immigrant)* ^47^

However, some Chinese immigrants believe that fulfilling filial obligations means respecting and supporting their parents’ choices about end-of-life care, even if it means limited or no use of life-sustaining treatment.^49^

*“When my mother was end of life, my brother and I knew what she wanted. But my mother’s siblings did not understand. They asked us ‘why didn’t you do more?’ This was so hurtful because if we did those things it would be against her wishes.”*

*(Young Chinese immigrant)* ^49^

1c. Differences in the interpretation of autonomy

Some Chinese immigrants were likely to rely on their families and tend to delegate the responsibility of making end-of-life care decisions to their families.^47^

*“Why bother to think of so many things?. . .. . . I don’t want to set up these responsibilities in my final days. I won’t appoint proxies. I will let my children do as they want. . . .”*

*(older Chinese immigrant)* ^47^

Other Chinese immigrants hope to include their family members in the advance care planning conversations.^51,54^

*“Once the patient has regained consciousness, the children should still discuss these options with the patient him/herself. Some people are unable to make decision on their own or are incompetent. In such instances, the children can help make decisions on their behalf.”*

*(older Chinese immigrant)* ^51^

Some other Chinese immigrants indicated that self-determination and the need for autonomy were consistent with their newly adopted cultural values.^39,43^

*“Our children need to know what we think. It’s our own business, our decision. Just tell them not to prolong [our life] or procrastinate [our death]”*

*(older Chinese immigrant)* ^43^

### Preferred approach to advance care planning

Our findings showed that Chinese immigrants would appreciate it if healthcare professionals initiate advance care planning discussions implicitly, contextualize its concept in Chinese culture, and use the Chinese language to enable rich, meaningful conversations.

2a. The use of an implicit approach

Chinese immigrants often appreciated that healthcare professionals use implicit communication strategies during advance care planning conversations and initiate such conversations during clinical routines or community activities.^24,39,41–43,50^

*“Whatever the provider said, we have to tell the patient. Professionally we really have to stick to the authenticity of what the provider said. But sometimes the provider’s word is a little bit too harsh. We have a lot of words in Chinese that are a little bit more polite than just telling them that you are going to die.”*

*(Chinese-speaking medical interpreter)* ^42^

Various implicit approaches were identified to facilitate advance care planning conversations with Chinese immigrants. One example is showing respect for the cultural taboos of discussing topics such as death and dying, and asking for permission before approaching such topics.^39^

*“The easiest way to raise up this topic is to make sure the healthcare professionals show they understand the Chinese culture. You can say, ‘I know in Chinese culture, we are supposed not to talk about that. . .’”*

*(46-year-old Chinese immigrant)* ^39^

Another example is initiating advance care planning conversations with prompts (positive scenarios or relevant experiences of others).^38,39^

*“After you have reassured them that they’re really healthy, just say, “You’re healthy. . . Hopefully I’ll be as healthy as you when I get to that age. I wish you live to 100. Do you want to live to 100?” Maybe that’s one way to open it. To start off with something positive and move on to seeing whether they have given any thought to this.”*

*(Primary care professional)* ^38^

In the Chinese language, there are many euphemisms referring to death. The use of euphemisms or metaphors is one of the implicit approaches to soften the taboo on discussing death.^50^

*“When an older person wants to ‘go’ let him go and complete the last path of his life. Do not send him to a nursing home or apply tracheostomy. He will have to go someday, right? I will tell my children, not to apply tracheostomy on me when I go in future. I want to go fast when it is time for me to go.”*

*(Older Chinese immigrant)* ^50^

Integrating advance care planning conversations with clinical routines or as part of community activities is another way to implicitly initiate advance care planning conversations, which could alleviate discomfort with this topic.^39,41^

*“The doctor can say, ‘. . .we just want to have this in our record. Would you mind filling this (an advance directive) out, so we will know how to take care of you according to your wish?’ So there’s no harm, no hard feeling.”*

*(Older Chinese immigrant)* ^39^

2b. The initiation by non-family members

Advance care planning conversations may be inconsistent with the Chinese family’s understanding of maintaining family harmony. Initiating older parents to make their own decisions about future medical care may be seen as an attempt to evade caregiving obligations. Additionally, due to the strong death taboo, such discussions may bring negative emotions within the family. In this case, healthcare professionals who are not part of the family structure are expected to introduce conversations to the family.^38^

*“I would like for the provider to initiate it first and then we will follow up with the topic with the parents.”*

*(Young Chinese American)* ^38^

In the Chinese community, respected community leaders often serve as opinion leaders. As they begin to plan for their own end-of-life, other community members may follow.^41^

*“I did not think for too long as I needed it (the plot) anyway. Since the chairperson of our association purchased first, so a few of us followed, then we could be ‘neighbors’ to each other” (laugh).*

*(Older Chinese immigrant)* ^52^

2c. The contextualization of distant philosophies

The concept and philosophical core of advance care planning are not common in Chinese society and can be difficult for Chinese immigrants to understand. Therefore there is a need to contextualize the concept of advance care planning in Chinese cultural values.^42,52^

*“I always tell our members, in Chinese culture, early preparation (alluding to advance care planning) used to be exclusive to the rich and powerful. In ancient China, only the emperors got to select their gravesites, to pre-arrange their funerals and to make wills. The ordinary peasant could not do it. So I asked my members, do you want to be an emperor or a peasant? (laugh).”*

*(the chairwoman of a Chinese health support service)* ^52^

2d. The use of Chinese language

Some Chinese immigrants reported being more comfortable using their native language during advance care planning conversations, despite their sufficient local language ability. Likewise, written information on advance care planning was better received when it was delivered in Chinese.^24,41^

*“I think discussing [ACP] in Chinese is definitely more comfortable. No matter how good your English is. . .”*

*(Older Chinese immigrant)* ^41^

Chinese immigrants reported the lack of detailed educational materials of advance care planning in Chinese that can guide Chinese immigrants on their end-of-life practices in the host country.^24^

## Discussion

Our review showed that Chinese immigrants differ in their willingness to engage in advance care planning. We found that Chinese immigrants’ acculturation influences their perceptions of their cultural identity and their interpretation of filial piety and autonomy. These various interpretations further influenced their perceptions of advance care planning and whether and how they would engage in it. Many Chinese immigrants prefer to have advance care planning conversations initiated by non-family members, in community activities, or clinical routines. Advance care planning conversations require the use of culturally sensitive communication skills to deliver the information implicitly, for instance by using euphemisms or metaphors. Properly contextualizing the concept of advance care planning in the Chinese culture is critical to improving its understanding.

Our findings suggest that Chinese immigrants’ cultural identity influences their willingness to engage in advance care planning. Being aware that immigrants’ cultural identity influences their interpretation of health-related messages from the host society, can help us understand their health-related behaviors.^55^ Recent evidence shows that acculturation, mostly capturing immigrants’ birthplace and the duration of their stay in the host country, is associated with their health-related behaviors and health outcomes.^56,57^ Chinese immigrants’ acculturation influences chronic condition management in a complex way, which is shaped by various factors, such as their cultural background, their goals for acculturation, and the pressure they feel to assimilate.^58^ The difference in their behavior indicates how Chinese immigrants understand the difference between cultural and societal norms and the strategies they took to manage this challenge.^59,60^ Wang and Yu reported that Chinese immigrants who identify with the American culture and purposely maintain interactions with this host culture are more likely to seek health information and use the healthcare services of their host society.^61^ Being more familiar with the medical norms in their host country, Chinese immigrants may correspondingly gain greater awareness and knowledge of advance care planning by being approached to discuss their preferences and plan for the future.^62^ In our study, individuals’ perception on which culture they belong to, influences which approach they would prefer for advance care planning. Understanding this would facilitate healthcare professionals in determining the direction to take when approaching Chinese immigrants for culturally sensitive advance care planning.

Our findings show that the concepts of filial piety and autonomy are highly relevant to Chinese immigrants’ willingness to engage in advance care planning. We found that for some Chinese immigrants, filial piety means behaving in a certain way to meet the need for collective identification and social expectations, while for others, filial piety manifests itself in understanding and supporting parents’ end-of-life wishes. According to the dual filial piety model,^63^ filial piety is interpreted differently across cultural societies in terms of the need for intimate relationships with parents and the need to fulfill social expectations.^64^ Both ways are often intertwined in practice.^65^ Cross-cultural studies of the conceptualization of filial piety found no difference in the needs for intimacy with parents in people from Asian countries and Western countries, and people from Western countries showed lower needs to fulfill social expectations than those from Asian countries.^64,66^ The fear of societal criticism for not exhibiting adequate care toward older parents, which is often prompted by the decision to withhold life-sustaining treatment, impacts individuals’ perspectives on advance care planning.^67^ In our findings, the idea of patient autonomy appeared to be important to some Chinese immigrants, but for many other Chinese immigrants, it did not resonate with their daily life experiences nor was it their primary consideration. In Chinese culture, autonomy may manifest itself at the level of collective decision-making unit, such as the family, rather than the individual patient.^68^ Harmony is the key value within the collective unit, which places a high value on interdependence and avoiding conflict.^19^ Therefore, medical decisions are often made with the collective interest of the family in mind.^69^ Healthcare professionals need to understand that there is not only one means of achieving and respecting patients’ autonomy. Understanding patients’ priorities in advance care planning and observing the family dynamics of patients’ decision-making process requires education for healthcare professionals.

We found that oftentimes, an implicit approach including euphemisms and metaphors was adopted in advance care planning conversations with Chinese immigrants. Euphemisms and metaphors play a valuable role in serious illness conversations, by delivering painful truth in a more gentle and comforting language.^70,71^ The potential role of using an implicit approach in serious illness communication is sometimes controversial.^72,73^ Patients with serious illnesses may appreciate an implicit approach from healthcare professionals, especially when they are not ready for the disturbing truth.^74,75^ Also, healthcare professionals use an implicit approach more often when they find it difficult to respond to patients’ emotional statements and uncertainties of prognosis.^76^ There are concerns that an implicit approach may lead to misinformation and confusion and may have a negative impact on patient decision-making.^76–78^ In our findings, medical interpreters sometimes acted as cultural brokers for explaining cultural nuances in specific cultural contexts. In clinical practice, medical interpreters’ role is seen as multifaceted, not only in facilitating the exchange of information, but more importantly, as facilitators of understanding and cultural mediators.^79–81^ In situations where patients have limited language proficiency, medical interpreters can play a crucial role. However, research suggests that interpreters often face challenges when balancing strict interpretation with cultural brokering.^42,82^ Therefore, we recommend that healthcare professionals understand and value their role in cross-cultural conversations to minimize cultural conflicts and increase mutual understanding. More effort should be made to explore how to reach the balance between interpretation accuracy and cultural sensitivity in the general communication process.

Our review showed that the Chinese community plays an important role in engaging Chinese immigrants in advance care planning. Its safe cultural environment and interactions with peers may provide a trusted context for advance care planning for Chinese immigrants. This echoes the experience that the recruitment, engagement, and retention of Chinese immigrants in healthcare initiatives can be enhanced by taking a relational approach and cultivating community relationships.^83,84^ The value of a community-based approach is that participants may discuss the information gained in the community activity, which can help to reduce stigma and misinformation surrounding important healthcare topics.^85^ This puts community leaders in a key position in promoting new ideas and facilitating health behaviors in the community. In our findings, Chinese community residents often showed trust in community leaders to obtain information related to advanced care planning. Further research should consider a collaborative community approach to advance care planning, including exploring the possible role of Chinese community leaders.

## Strengths and limitations

To the best of our knowledge, this is the first systematic review to synthesize available evidence on the role of acculturation in Chinese immigrants’ willingness to engage in advance care planning. We utilized a comprehensive acculturation framework and elements to provide an in-depth understanding of the process and its effect. A dual coding was conducted independently by two authors (TZ, DM) to reduce the deviation of the interpretation of each research. We also included multiple perspectives: Chinese immigrants (older adults, patients, family members, Chinese community leaders) and healthcare professionals who care for them (clinicians, nurses and medical interpreters).

In interpreting the results of this systematic review, several limitations should be taken into account. First, the majority of the studies we included were conducted in community settings, which may lack generalizability to clinical settings. Second, we included only studies conducted in countries ranked in the top 25 of the 2015 Quality of Death Index,^30^ this may limit the generalization of our findings to Chinese immigrants residing in countries with lower quality of death rankings. However, we found no studies conducted in countries or regions outside of the top 25. The majority of included studies were from high-income Western countries, which may limit the generalization of these findings to countries with different cultural and socioeconomic development. Lastly, due to the limitation of data, we were unable to summarize which generation of Chinese immigrants our data originated from.

## Conclusion

The acculturation of Chinese immigrants influences their interpretation of autonomy and filial piety, as well as perceptions of whether and how advance care planning is compatible with their cultural identity. It is important to raise awareness among healthcare professionals about the diversity of Chinese immigrants in engaging in advance care planning, and the significance of carefully assessing Chinese immigrants’ values related to advance care planning. Strategies to promote advance care planning among Chinese immigrants may include implicitly initiating and discussing advance care planning, promoting a shared understanding of advance care planning among family members, and an interactive community-based approach. Further research is needed to explore how acculturation influences various aspects of advance care planning, such as the sharing and conversation process. In addition to considering birthplace and residency duration in the host country, it would be beneficial to incorporate additional dimensions of acculturation in the research process and investigate their association with health behaviors.

## Supplemental Material

sj-pdf-1-pmj-10.1177_02692163231179255 – Supplemental material for The role of acculturation in the process of advance care planning among Chinese immigrants: A narrative systematic review Click here for additional data file. Supplemental material, sj-pdf-1-pmj-10.1177_02692163231179255 for The role of acculturation in the process of advance care planning among Chinese immigrants: A narrative systematic review by Tingting Zhu, Diah Martina, Agnes van der Heide, Ida J Korfage and Judith AC Rietjens in Palliative Medicine

sj-pdf-2-pmj-10.1177_02692163231179255 – Supplemental material for The role of acculturation in the process of advance care planning among Chinese immigrants: A narrative systematic review Click here for additional data file. Supplemental material, sj-pdf-2-pmj-10.1177_02692163231179255 for The role of acculturation in the process of advance care planning among Chinese immigrants: A narrative systematic review by Tingting Zhu, Diah Martina, Agnes van der Heide, Ida J Korfage and Judith AC Rietjens in Palliative Medicine
