# Supplementary material for: The role of acculturation in the process of advance care planning among Chinese immigrants: A narrative systematic review
Source: Palliat Med. 2023 Jun 13;37(8):1063–78. doi: 10.1177/02692163231179255 (PMC10503260; doi:10.1177/02692163231179255)
Supplement: sj-pdf-1-pmj-10.1177_02692163231179255 – Supplemental material for The role of acculturation in the process of advance care planning among Chinese immigrants: A narrative systematic review [file sj-pdf-1-pmj-10.1177_02692163231179255.pdf]

Table 1 Search strategy

**Embase**	('living will'/exp OR 'attitude to death'/exp OR (('patient decision making'/exp OR 'decision making'/de OR 'interpersonal communication'/exp OR 'doctor patient relation'/de OR 'patient information'/de OR 'patient preference'/de OR 'patient autonomy'/de OR 'personal autonomy'/de OR 'patient attitude'/de OR 'knowledge'/exp OR 'personal experience'/de) AND ('terminal care'/exp OR 'palliative therapy'/exp OR 'terminally ill patient'/exp OR 'terminal disease'/de OR 'life threat'/exp OR 'advanced cancer'/de OR resuscitation/de OR 'life sustaining treatment'/de OR euthanasia/de OR hospice/de)) OR (((Advance) NEAR/3 (plan* OR directive*)) OR ((living-will*)) OR ((decision* OR decid* OR plan OR plans OR planning OR preference* OR want OR wish* OR dilemma* OR refus* OR choos* OR choice* OR communication OR talking OR disclos* OR autonom* OR attitude* OR pratice* OR perspective*) NEAR/6 (terminal* OR 'end of life' OR palliativ* OR serious*-ill* OR severe*-ill* OR death OR dying OR advanced*-cancer* OR euthanas* OR hospice*)) OR ((do-not OR refus*) NEAR/3 resuscit*) OR ((decision* OR decid* OR plan OR plans OR planning OR preference* OR want OR wish* OR dilemma* OR refus* OR choos* OR choice* OR communication OR talking OR disclos* OR autonom* OR attitude* OR pratice* OR perspective*) NEAR/6 (life-saving OR life-saver* OR life-sustain* OR life-resuscit* OR life-threat* OR life-support*))):ab,ti,kw) **AND** ((('migrant'/exp OR 'migration'/exp OR 'Australia and New Zealand'/exp OR 'Australian'/exp OR 'New Zealander'/de OR 'North America'/exp OR 'Europe'/de OR 'Western Europe'/exp OR 'Italy'/exp OR 'Spain'/exp OR 'Portugal'/exp OR 'Austrian'/de OR 'German (citizen)'/de OR 'Western European'/exp OR 'Northern European'/exp OR 'EU citizen'/de OR 'Italian (citizen)'/de OR 'Portuguese (citizen)'/de OR 'Spaniard'/de OR 'European'/de OR 'North American'/exp) AND ('Chinese'/exp OR 'China'/exp OR 'Far East'/de OR 'Asia'/de OR 'Asian'/de OR 'East Asian'/de OR 'Asian continental ancestry group'/exp)) OR 'Asian American'/exp OR 'British Asian'/exp OR (((china* OR chines* OR asia* OR arabia* OR orient OR hong-kong* OR beijing* OR far-east*) NEAR/6 (immigrant* OR migrant* OR emigrant* OR migrat* OR europe* OR european* OR scandinavia* OR america* OR US OR U-S OR united-state* OR canada* OR canadian* OR australia* OR england* OR UK OR U-K OR united-kingdom* OR british OR britain OR english OR scotland* OR scottish OR scotsman OR new-zealand* OR ireland* OR irish* OR belgium* OR belgian OR german* OR netherland* OR dutch* OR holland* OR france* OR french* OR norway* OR norwegian* OR switzerland* OR swiss* OR sweden* OR swedish* OR austria* OR denmark* OR danish* OR finland* OR finn* OR italy* OR italian* OR spain* OR spanish* OR spaniard* OR portugal* OR portuguese*))):ab,ti,kw)
**Medline**	(Living Wills/ OR Attitude to Death/ OR ((exp Decision Making/ OR exp Communication/ OR Physician-Patient Relations/ OR Patient Preference/ OR Personal Autonomy/ OR Knowledge/) AND (exp Terminal Care/ OR Palliative Care/ OR exp Resuscitation/ OR Hospices/)) OR (((Advance) ADJ3 (plan* OR directive*)) OR ((living-will*)) OR ((decision* OR decid* OR plan OR plans OR planning OR preference* OR want OR wish* OR dilemma* OR refus* OR choos* OR choice* OR communication OR talking OR disclos* OR autonom* OR attitude* OR pratice* OR perspective*) ADJ6 (terminal* OR end of life OR palliativ* OR serious*-ill* OR severe*-ill* OR death OR dying OR advanced*-cancer* OR euthanas* OR hospice*)) OR ((do-not OR refus*) ADJ3 resuscit*) OR ((decision* OR decid* OR plan OR plans OR planning OR preference* OR want OR wish* OR dilemma* OR refus* OR choos* OR choice* OR communication OR talking OR disclos* OR autonom* OR attitude* OR pratice* OR perspective*) ADJ6 (life-saving OR life-saver* OR life-sustain* OR life-resuscit* OR life-threat* OR life-support*))).ab,ti,kf.) AND (((Transients and Migrants/ OR Human Migration/ OR exp North America/ OR Europe/ OR exp Australasia/ OR Austria/ OR Belgium/ OR exp France/ OR exp Germany/ OR Ireland/ OR exp Italy/ OR Netherlands/ OR Spain/ OR Switzerland/ OR Portugal/ OR exp Scandinavian and Nordic Countries/ OR exp United Kingdom/ OR European Continental Ancestry

	Group/) AND (exp Asian Continental Ancestry Group/ OR exp China/ OR Far East/ OR Asia/)) OR Asian Americans/ OR (((china* OR chines* OR asia* OR arabia* OR orient OR hong-kong* OR beijing* OR far-east*) ADJ6 (immigrant* OR migrant* OR emigrant* OR migrat* OR europe* OR european* OR scandinavia* OR america* OR US OR U-S OR united-state* OR canada* OR canadian* OR australia* OR england* OR UK OR U-K OR united-kingdom* OR british OR britain OR english OR scotland* OR scottish OR scotsman OR new-zealand* OR ireland* OR irish* OR belgium* OR belgian OR german* OR netherland* OR dutch* OR holland* OR france* OR french* OR norway* OR norwegian* OR switzerland* OR swiss* OR sweden* OR swedish* OR austria* OR denmark* OR danish* OR finland* OR finn* OR italy* OR italian* OR spain* OR spanish* OR spaniard* OR portugal* OR portuguese*))).ab,ti,kf.)
**Cochrane**	((((Advance) NEAR/3 (plan* OR directive*)) OR (living NEXT/1 will) OR ((decision* OR decid* OR plan OR plans OR planning OR preference* OR want OR wish* OR dilemma* OR refus* OR choos* OR choice* OR communication OR talking OR disclos* OR autonom* OR attitude* OR pratice* OR perspective*) NEAR/6 (terminal* OR 'end of life' OR palliativ* OR (serious* NEXT/1 ill*) OR (severe* NEXT/1 ill*) OR death OR dying OR (advanced* NEXT/1 cancer*) OR euthanas* OR hospice*)) OR (((do NEXT/1 'not') refus*) NEAR/3 resuscit*) OR ((decision* OR decid* OR plan OR plans OR planning OR preference* OR want OR wish* OR dilemma* OR refus* OR choos* OR choice* OR communication OR talking OR disclos* OR autonom* OR attitude* OR pratice* OR perspective*) NEAR/6 ((life NEXT/1 saving) OR (life NEXT/1 saver*) OR (life NEXT/1 sustain*) OR (life NEXT/1 resuscit*) OR (life NEXT/1 threat*) OR (life NEXT/1 support*)))):ab,ti,kw) **AND** ((((china* OR chines* OR asia* OR arabia* OR orient OR (hong NEXT/1 kong*) OR beijing* OR (far NEXT/1 east*)) NEAR/6 (immigrant* OR migrant* OR emigrant* OR migrat* OR europe* OR european* OR scandinavia* OR america* OR US OR (U NEXT/1 S) OR (united NEXT/1 state*) OR canada* OR canadian* OR australia* OR england* OR UK OR (U NEXT/1 K) OR (united NEXT/1 kingdom*) OR british OR britain OR english OR scotland* OR scottish OR scotsman OR (new NEXT/1 zealand*) OR ireland* OR irish* OR belgium* OR belgian OR german* OR netherland* OR dutch* OR holland* OR france* OR french* OR norway* OR norwegian* OR switzerland* OR swiss* OR sweden* OR swedish* OR austria* OR denmark* OR danish* OR finland* OR finn* OR italy* OR italian* OR spain* OR spanish* OR spaniard* OR portugal* OR portuguese*))):ab,ti,kw)
**Web of Science**	TS=(((((Advance) NEAR/2 (plan* OR directive*)) OR (((living NEAR/1 will*))) OR ((decision* OR decid* OR plan OR plans OR planning OR preference* OR want OR wish* OR dilemma* OR refus* OR choos* OR choice* OR communication OR talking OR disclos* OR autonom* OR attitude* OR pratice* OR perspective*) NEAR/5 (terminal* OR "end of life" OR palliativ* OR serious*-ill* OR severe*-ill* OR death OR dying OR advanced*-cancer* OR euthanas* OR hospice*)) OR ((do-not OR refus*) NEAR/2 resuscit*) OR ((decision* OR decid* OR plan OR plans OR planning OR preference* OR want OR wish* OR dilemma* OR refus* OR choos* OR choice* OR communication OR talking OR disclos* OR autonom* OR attitude* OR pratice* OR perspective*) NEAR/5 life NEAR/1 (saving OR saver* OR sustain* OR resuscit* OR threat* OR support*)))) AND ((((china* OR chines* OR asia* OR arabia* OR orient OR hong-kong* OR beijing* OR far-east*) NEAR/5 (immigrant* OR migrant* OR emigrant* OR migrat* OR europe* OR european* OR scandinavia* OR america* OR US OR U-S OR united-state* OR canada* OR canadian* OR australia* OR england* OR UK OR U-K OR united-kingdom* OR british OR britain OR english OR scotland* OR scottish OR scotsman OR new-zealand* OR ireland* OR irish* OR belgium* OR belgian OR german* OR netherland* OR dutch* OR holland* OR france* OR french* OR norway* OR norwegian* OR switzerland* OR swiss* OR sweden* OR swedish* OR austria* OR denmark* OR danish* OR finland* OR finn* OR italy* OR italian* OR spain* OR spanish* OR spaniard* OR portugal* OR portuguese*)))))

**Google Scholar**	"living will|wills"|"attitude|preference|decision|planning|wish|dilemma death|euthanasia|terminal|hospice"|"advanced care planning" "migrant|migration|europe|america|australia china|Chinese|asia"
