# Supplementary material for: The role of acculturation in the process of advance care planning among Chinese immigrants: A narrative systematic review
Source: Palliat Med. 2023 Jun 13;37(8):1063–78. doi: 10.1177/02692163231179255 (PMC10503260; doi:10.1177/02692163231179255)
Supplement: sj-pdf-2-pmj-10.1177_02692163231179255 – Supplemental material for The role of acculturation in the process of advance care planning among Chinese immigrants: A narrative systematic review [file sj-pdf-2-pmj-10.1177_02692163231179255.pdf]

Supplementary table 2: Quality assessment scores for included qualitative studies

	Author, year (Host country)	Objective	Study design	Study Context	Theoretical	Sampling strategy	Data collection	Data analysis	Verification,	Results and Conclusio	Reflexivity	Total Sum	Summary Score	Quality
1	KL Braun,1996 (the United State) [41]	1	2	1	0	1	1	0	2	2	0	10	0.50	Limited
2	KL Braun,1997 (the United State)[36]	1	2	1	0	1	1	0	2	2	0	10	0.50	Limited
3	KW Bowman, 2001 (Canada)[43]	2	2	1	0	2	2	1	2	2	0	14	0.70	Adequate
4	AG Yick, 2002 (the United State)[40]	2	2	1	0	1	1	1	0	2	0	10	0.50	Limited
5	J Chan, 2005 (the United State)[42]	2	2	2	0	1	1	1	0	2	0	11	0.55	Adequate
6	G Bellamy, 2013 (New Zealand)[50]	2	2	2	2	2	2	1	0	2	0	15	0.75	Good
7	ML Fang, 2015 (the United Kingdom)[44]	2	2	2	2	2	1	2	0	2	2	17	0.85	Strong
8	LS Nielsen, 2015 (Canada)[49]	2	2	1	2	2	1	1	1	2	0	14	0.70	Good
9	J Yonashiro-Cho, 2016 (the United State)[39]	2	2	1	2	2	1	2	0	2	0	14	0.70	Strong
10	MC Lee, 2017 (the United State)[37]	2	2	1	1	2	2	2	2	2	0	16	0.80	Good
11	SS Yap, 2017 (Australia)[47]	2	2	2	0	2	2	2	2	2	0	16	0.80	Good
12	HL Chi, 2018 (the United State)[34]	2	2	2	2	2	1	1	2	2	0	16	0.80	Good
13	HL Chi, 2018 (the United State)[35]	2	2	2	2	2	1	1	2	2	0	16	0.80	Good
15	MD Silva, 2020 (the United State)[38]	2	2	2	0	2	1	2	2	2	0	15	0.75	Good

16	WY Wang, 2020 (Australia)[48]	2	2	2	2	2	2	1	2	2	0	17	0.85	Strong
17	W Zhang, 2020 (Canada)[46]	2	2	1	2	1	2	2	0	2	0	14	0.70	Adequate

Supplementary table 3: Quality assessment scores for included quantitative studies

	Author, year (Host country)	Objective	Study design	Subject selection	Subject characteristics	Random allocation	blinding of investigators	blinding of subjects	Outcome exposure	Sample size	Analysis methods	estimate of variance	Controlled for confounding	Results	Results and conclusion	Total Sum	Summary Score	Quality
1	X Gao, 2015 (the United States)[15]	2	2	2	2	N/A	N/A	N/A	2	2	1	2	2	1	2	20	0.90	Strong
2	L Dhingra, 2020 (the United States)[20]	2	2	2	2	N/A	N/A	N/A	2	1	2	1	2	2	0	18	0.82	Strong
3	Y Pei, 2021 (the United States)[22]	2	2	2	1	N/A	N/A	N/A	1	1	2	2	2	1	2	18	0.82	Strong
4	K Wang, 2021 (the United States)[21]	2	2	2	2	N/A	N/A	N/A	2	1	2	2	2	1	2	20	0.90	Strong

N/A: not applicable
